# Forecasting demand data for critical materials

**DOI:** 10.1016/j.dib.2024.111013

**Published:** 2024-10-09

**Authors:** Kyle Foster, Nehika Mathur

**Affiliations:** a Miami University, 501 E. High Street, Oxford, OH 45056, United States; b National Institute of Standards & Technology, 100 Bureau Drive, Gaithersburg, MD 20899, United States

**Keywords:** Bass diffusion model, Market dynamics, Cobalt, gallium, Indium, critical materials

## Abstract

Gallium, Indium and Cobalt are critical materials that are vital to ramping up the adoption of clean energy technologies such as solar photovoltaics (PVs), electric vehicles (EVs) and wind turbines (WTs). Like other critical materials, they are prone to supply chain risks resulting in future uncertainty associated with supply, demand and price parameters. Understanding current and future market dynamics will mitigate this uncertainty. This article applies the Bass Diffusion Model to generate demand projections as three data sets each for Gallium, Indium and Cobalt until 2050. Generating and compiling these data are one step in enabling insights on future market parameters of the considered materials and the clean energy products that rely on their availability. Going forward this data has the potential to contribute towards understanding other market parameters such as supply and prices.

Specifications Table

**Table:** 

Subject	Sustainability and the Environment, Engineering
Specific subject area	Sustainability and the Environment, and modelling market dynamics
Type of data	Data tables Graphs Python scripts
Data collection	Bass parameters were computed and subsequently the Bass Diffusion Model was applied to forecast global demands for critical materials, Gallium, Indium and Cobalt [[1], [2], [3], [4]]. Raw data comprising historical production data was accessed via The British Geological Survey [5]*.*
Data source location	National Institute of Standards and Technology, Gaithersburg, MD 20,899, USA
Data accessibility	*The data is publicly available online (see below)* Repository name: NIST Management of Institutional Data Assets (MIDAS) Data identification number: https://data.nist.gov/od/id/mds2–3442 Direct URL to data: https://data.nist.gov/od/id/mds2–3442 Persistent Identifier: doi:10.18434/mds2–3442
Related research article	Mathur, N., Maani, T., Rong, C., & Sutherland, J. W. (2024). Forecasting Rare Earth Element Demands for Clean Energy Technologies with the Bass Diffusion Model. *Procedia CIRP, 122*, 55–60.

## 1 Value of the Data

• Per the Intergovernmental Panel on Climate Change (IPCC) Assessment report (AR) 6, there is a requirement for “deep, rapid and sustained global Greenhouse Gas (GHG) reduction this decade” [6]. Facilitating GHG reductions will require the transition to a decarbonized economy, which in turn will be enabled via the adoption of clean energy technologies such as Electric Vehicles (EVs), Wind Turbines (WTs) and Solar Photovoltaics (PVs).
• The above-mentioned clean energy technologies rely on the steady supply of critical materials. According to the United States, Department of Energy, critical materials are those materials that are susceptible to supply chain risks and find applications in energy technologies that produce, transmit, store or conserve energy. [3]. The current risks associated with many critical material supply chains, can jeopardize the adoption of clean energy technologies. There is therefore a need to be able to anticipate changing market conditions to mitigate these uncertainties and ensure the steady supply of critical materials.
• The first step in addressing market uncertainties is understanding current and historical material demands. Unfortunately, when it comes to critical materials, there is very limited market-specific data available. Understanding the demand trends for critical materials has the potential to provide insights via predictive modeling methods into other market parameters such as future pricing structures. The novelty of this work lies in applying a forecasting model, the Bass Diffusion Model to determine future demands of three critical materials, namely, Gallium (Ga), Indium (In) and Cobalt (Co). The demand datasets presented in this work address a major gap in the scientific literature.
• The datasets generated will provide other researchers a range of anticipated consumption trends for the identified materials until 2050. This is turn will help researchers identify suitable primary supply sources, drive the development of secondary supply sources and help strategize the production of clean energy products that use these materials.
• It is anticipated that these datasets, namely, demand estimates for Ga, In and Co will be useful to variety of stakeholders. Besides scientific researchers and economists, clean energy technology manufacturers, such as those producing EVs, WTs and PVs will benefit from gaining an understanding of overall future feedstock demands. Additionally, governments keen to enable the decarbonization of their respective economies will benefit from knowing what the future critical material demands are to work towards developing strategies to either meet these demands by implementing measures to secure supply chains, such as identifying suitable substitute materials and developing secondary sources.

## 2 Background

**Fig. 1:**
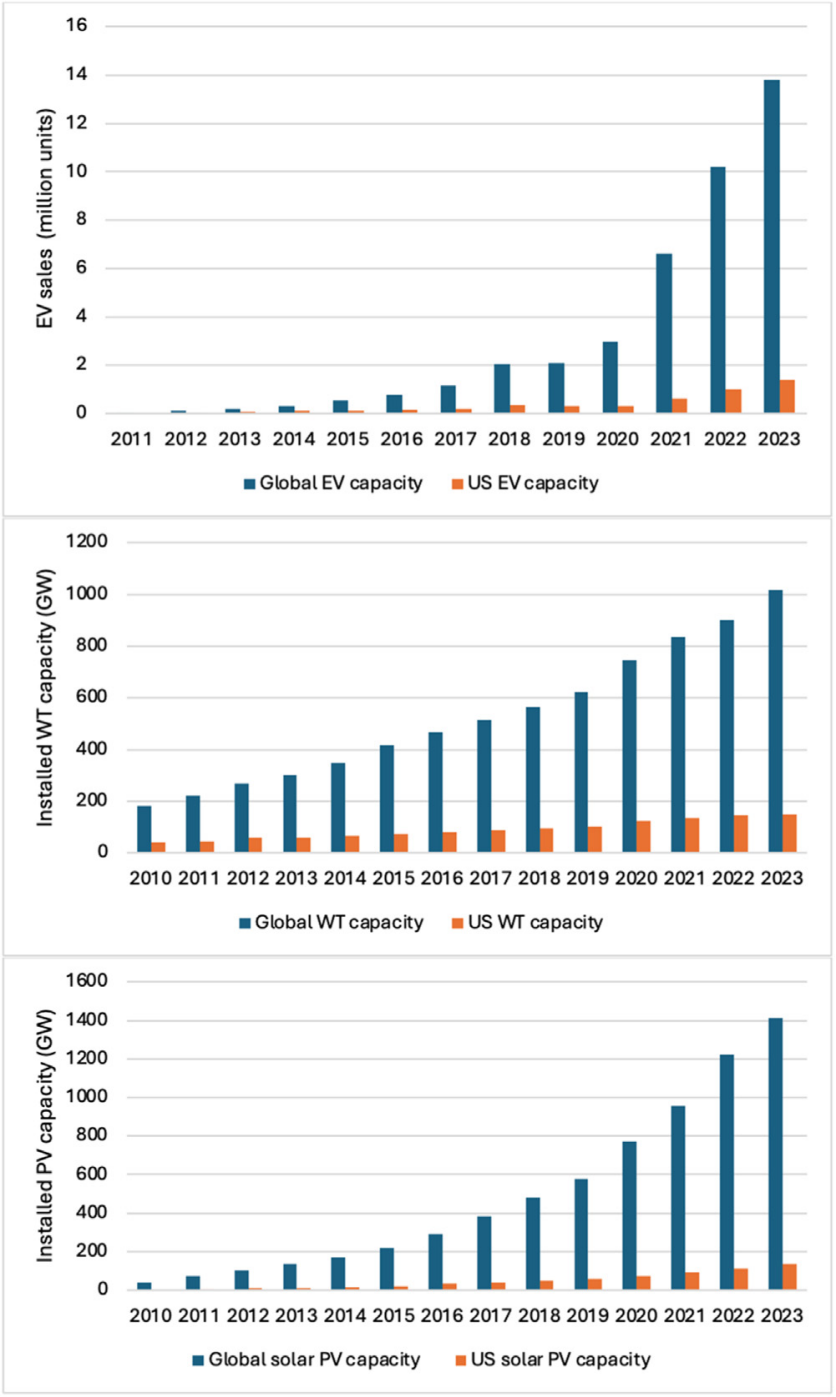
Historical clean energy technologies growth (2010 - 2023) adapted from [1,2]. The figures above reiterate the strong growth in the clean energy technology sector, globally and across the US. (Installed PV and WT capacity refers to the maximum amount of energy that can be generated under ideal conditions).

Clean energy technologies such as PVs, EVs and WTs are vital in the transition to a decarbonized energy grid. Their growth in the recent past has been unprecedented (Fig. 1). Many clean energy technologies including PVs, EVs and WTs rely on critical materials that are susceptible to supply chain risks. For instance, PVs solar panels use Ga and In. Ga also find application in offshore WTs along with Co. Both Ga and Co are also used in EVs as well as conventional internal combustion engine (ICE) vehicles (Table 1).

**Table 1:** An overview of clean energy products and components utilizing Ga, In and Co (in bold) [3].

Energy Generation	Nuclear	Fuels, moderators	U, Zr, natural graphite, electrical steel

	**Solar**	**PVs**	Si, Te, **Ga, In**
	**Wind**	**Off-shore**	Cu, Nd, Pr, Dy, Tb, B, **Ga, Co**, electrical steel
		Land-based	Cu, electrical steel

**Energy storage**	**Fuel cells**	**Stationary hydrogen to electricity conversion**	Pt, graphite, La, Sr, **Co**, Ni, Y, Zr, Mn
	**Batteries**	**Lithium-ion batteries, zinc air, iron air, sodium air, flow batteries**	Li, **Co**, Ni, graphite, V, Zn, Fe, Al, Na, S, P, F

**End-use**	**Lighting**	LEDs*	**Ga**
	**Consumer electronics**	**Power electronics**	**GaN**, SiC
	**Electric vehicles**	Power electronics	SiC
		Lightweighting	Mn, Mg, Al, Ni, Si
		**Magnets**	Nd, Pr, Dy, Tb, B, Fe, **Ga**
		**Batteries**	Li, Ni, Mn, Co (in NMC*batteries), graphite, Al, Fe, P, LREEs*
		Motors	electrical steel, Cu
		Wiring	Cu
	Optoelectronics	Microchips	Ge
	**Vehicles**	Lightweighting	Mn, Mg, Al, Ni, Si
		Catalysts	Pt, Pd, Rh
		Motors	electrical steel, Cu
		**Fuel cells**	Pt, graphite, La, Sr, **Co**, Ni, Y, Zr, Mn
		Wiring	Cu
	**Hydrogen**	**Hydrogen electrolyzers**	Pt, Ir, Ti, La, Sr, **Co**, Ni, Y, Zr, Mn

⁎ LED = Light emitting Diode, NMC = Nickel- Manganese-Cobalt, LREE = Light Rare Earth Element.

As the demand for PVs, EVs and WTs grows so will the demand for Ga, In and Co [1]. The Bass Diffusion Model is applied to forecast demands for Ga, In and Co. The Bass coefficients of innovation, p and imitation, q was computed from raw data and applied to Eq (1) to obtain demand projections until 2050. Future research could use these demand projections to determine other market parameters that could help influence and shape supply chains. The novelty of this work is that it applies the Bass Diffusion Model to forecast material demands for critical materials, namely, Ga, In and Co, thereby addressing a major data gap in literature.

## 3 Data Description

This data set comprises Bass Diffusion Model coefficients, p & q (computed using the historical data determined from literature) for Ga, In and Co. Three demand scenarios for different values of market size, N were investigated (Table 2).

**Table 2:** An overview of the Bass parameters used (N, p & q). The market size, N was obtained from literature [1]. The coefficients p and q were computed using historical demand data in literature [5].

Critical material	Demand scenario	N (million units)	p	q
**Ga**	Low	33	2.46E-06	0.130
	Mean	50		
	High	67		
**In**	Low	33	1.23E-05	0.252
	Mean	50		
	High	67		
**Co**	Low	33	7.13E-04	0.071
	Mean	50		
	High	67		

**Fig. 2:**
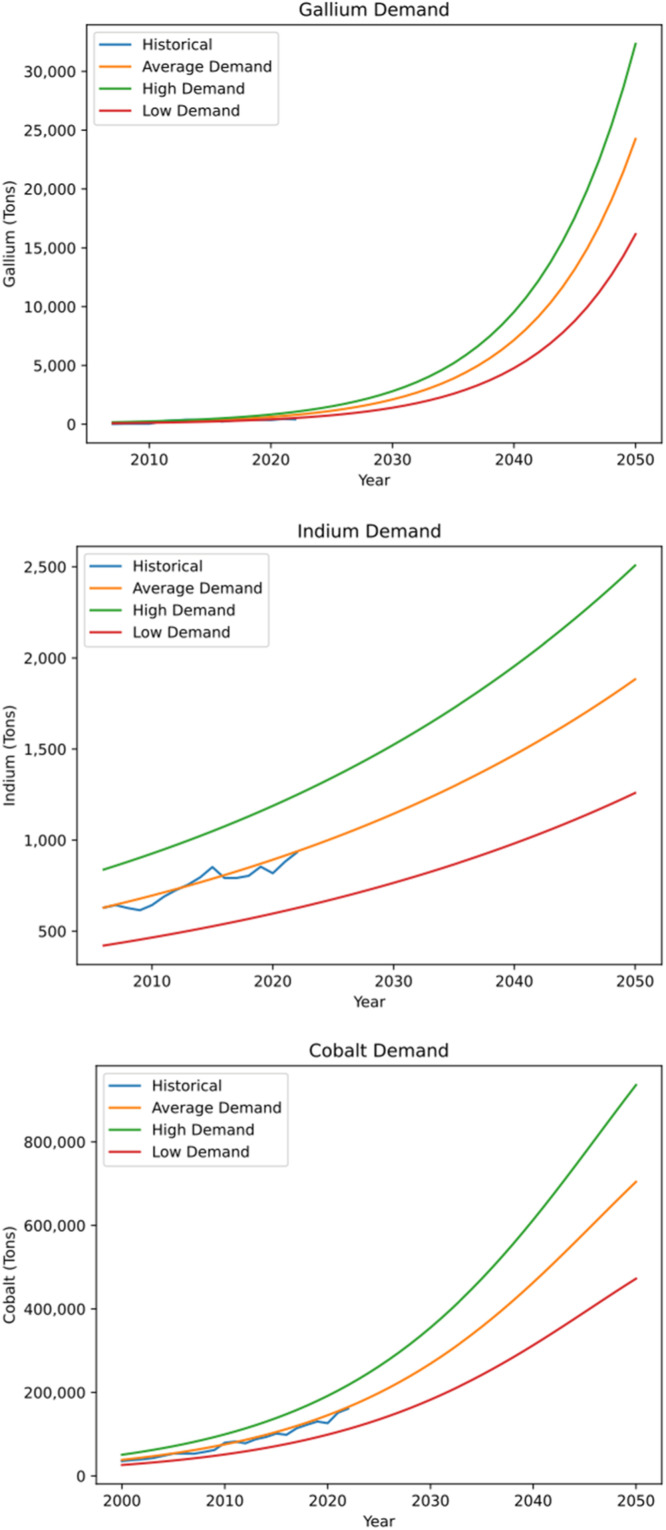
Ga, In and Co Bass Diffusion Model global demand trends until 2050 under low (red), mean (orange) and high (green) market size scenarios. The historical demand data obtained from literature is represented in blue [5].

The generated data set comprises global demand data for critical materials, Ga, In and Co for average, low and high demand scenarios based on varying market size, N until 2050 (Fig. 2). Disaggregated data is available online in the NIST repository [4] (Table 3).

**Table 3:** A summary of datasets in the linked repository [4]. Supporting data files comprise historical demand trends for Ga, In and Co, computed p and q values for Ga, In and Co, and the Bass Diffusion Model scripted in Python [4].

File name	Contents
BassModel_datacollection.xlsx	Tab 1: Historic global production of Cobalt (2000–2022 [5] Tab 2: Historic global production of Indium (2006–2022) [5] Tab 3: Historic global production of Gallium (2007–2022) [5]
BassModel_Gallium-2007–2050.xlsx	Tab 1: Historic global production data & computed Bass coefficients, p and q values for Ga Tab 2: Average, High and Low scenario demand values until 2050 for Ga
BassModel_Gallium-graph.pdf	Plotted historic production and demand values for Ga
BassModel_Gallium.ipynb	Jupiter notebook with Bass Model coded for Ga demand forecasts
BassModel_Indium-2006–2050.xlsx	Tab 1: Historic global production data & computed Bass coefficients, p and q values for In Tab 2: Average, High and Low scenario demand values until 2050 for In
BassModel_Indium-graph.pdf	Plotted historic production and demand values for In
BassModel_Indium.ipynb	Jupiter notebook with Bass Model coded for In demand forecasts
BassModel_Cobalt-2006–2050.xlsx	Tab 1: Historic global production data & computed Bass coefficients, p and q values for Co Tab 2: Average, High and Low scenario demand values until 2050 for Co
BassModel_Cobalt-graph.pdf	Plotted historic production and demand values for Co
BassModel_Cobalt.ipynb	Jupiter notebook with Bass Model coded for Co demand forecasts

## 4 Experimental Design, Materials And Methods

**Fig. 3:**
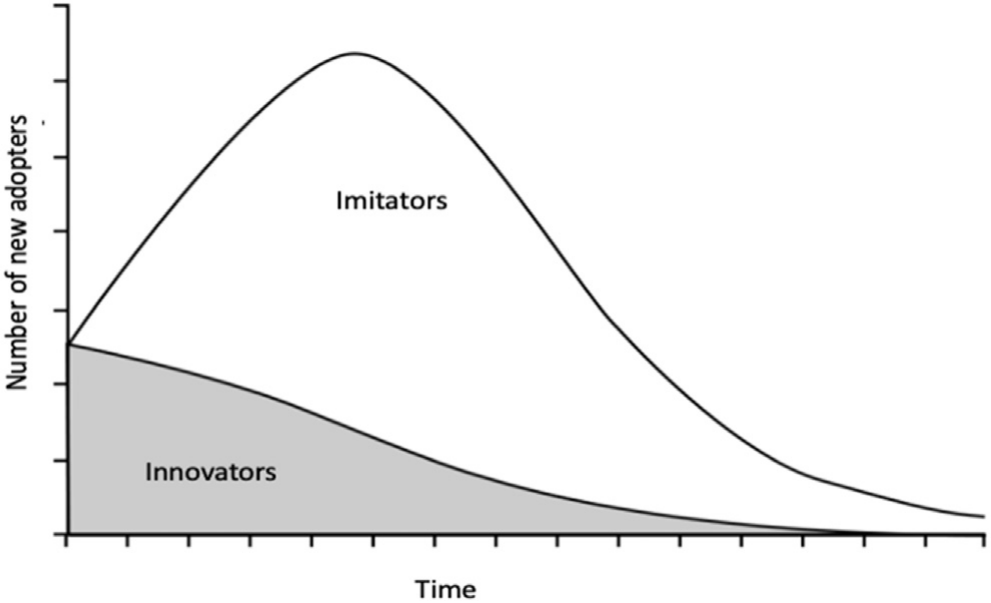
The Bass Diffusion Model - A representation of new adopters over time Bass Diffusion Model (adapted from [1,8]).

**Fig. 4:**
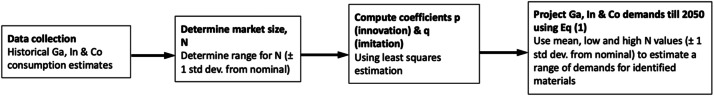
An overview of the steps involved in forecasting demands for Ga, In and Co until 2050.

This work applies the Bass Diffusion Model to forecast demand data for critical materials, Ga, In and Co. The Bass Diffusion Model is a mixed influence, generalized model that captures the impacts of both innovation and imitation on the diffusion process of a new product or material in a market. This is done by dividing the market consumers into two categories, namely innovators and imitators. Innovators are that segment of the market that remain uninfluenced by the actions of other individuals when it comes to acquiring a new product. Innovators gather information and base their decision to adopt a new product from mass media and advertisements. Imitators on the other hand, are those individuals in a market that are influenced by the others’ actions. They gather the information based on their decision to adopt a new product because of word-of-mouth and other informal feedback. The Bass model relies on the assumption that the impacts of advertising a new product is greater early on. Over time however, the impact of word-of-mouth communication becomes more significant in the diffusion process [7] (Fig. 3, Fig. 4).

The Bass Diffusion Model is mathematically represented via Eqs. (1)–(3).

(1) f(t)=(p+qF(t))(1−F(t))

(2) f(t)=a(t)/N

(3) F(t)=A(t)/N

Where,

*f*(*t*), density function of sales at time t
*a*(*t*), product purchase rate at time t
*F*(*t*), cumulative fraction of potential growth at time t
*A*(*t*), total number of adopters at time t
*N*, total number of potential buyers
p, coefficient of innovation
q, coefficient of imitation

SThe new product diffusion process depends on market size, N, and the coefficients of innovation and imitation, p & q respectively. Market size, N was based on values found in the literature [1]. The coefficients, p and q were computed from using historical Ga, In and Co data using least squares [4]. Subsequently, the Bass model was scripted in Python and applied to forecast global demands for all three materials until 2050 [4]. A range of demands for minimum and maximum values of N (± one standard deviation) has been generated.

## Limitations

Although the Bass Diffusion Model is advantageous in that it is a parsimonious model and requires only limited data, it does have some limitations in its current form. The Bass model does assume that every new product is a success and therefore its sales will reach a steady state. For instance, in the future, there could be numerous market-related events (e.g., introduction of novel technologies or substitute materials) that could potentially cause this prediction to be less accurate. While the model does take internal and external elements into account, it cannot predict the drastic shifts generated by technological advances or market disruptions thus resulting in uncertainty. The model does provide a useful starting point in estimating future demands for critical materials such as Ga, In and Co that are consumed in novel clean energy technologies, whose market dynamics themselves are uncertain. Greater accuracy can be achieved as more disaggregated data becomes available on individual material consumption patterns for a range of different applications. In its present form though and in the absence of disaggregated data, the authors have provided a range for the projected demands for the materials under consideration in this study.

## Ethics Statement

The authors have read and follow the ethical requirements for publication in Data in Brief and confirming that the current work does not involve human subjects, animal experiments, or any data collected from social media platforms.

## CRediT authorship contribution statement

**Kyle Foster:** Methodology, Validation, Software, Formal analysis, Investigation, Writing – original draft, Visualization. **Nehika Mathur:** Conceptualization, Methodology, Software, Validation, Formal analysis, Investigation, Writing – original draft, Visualization, Project administration, Supervision.

## Data Availability

NIST Public Data RepositoryForecasting demand data for critical materials (Original data).
